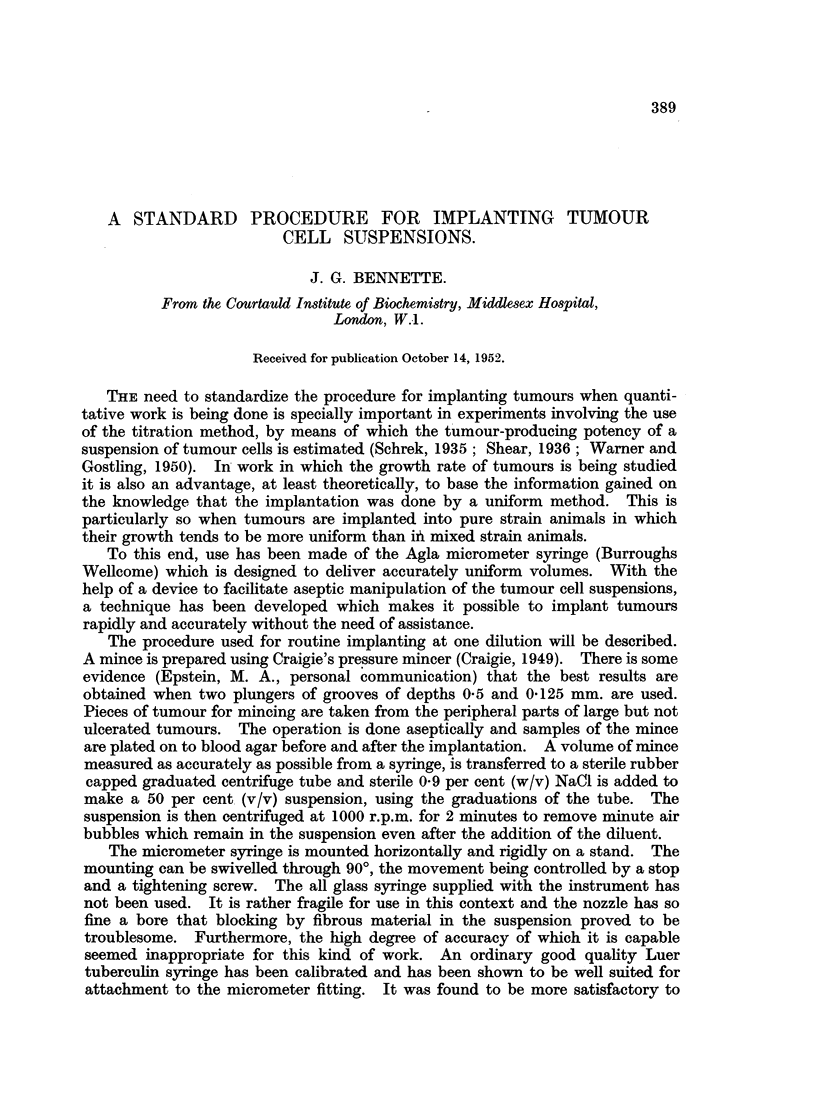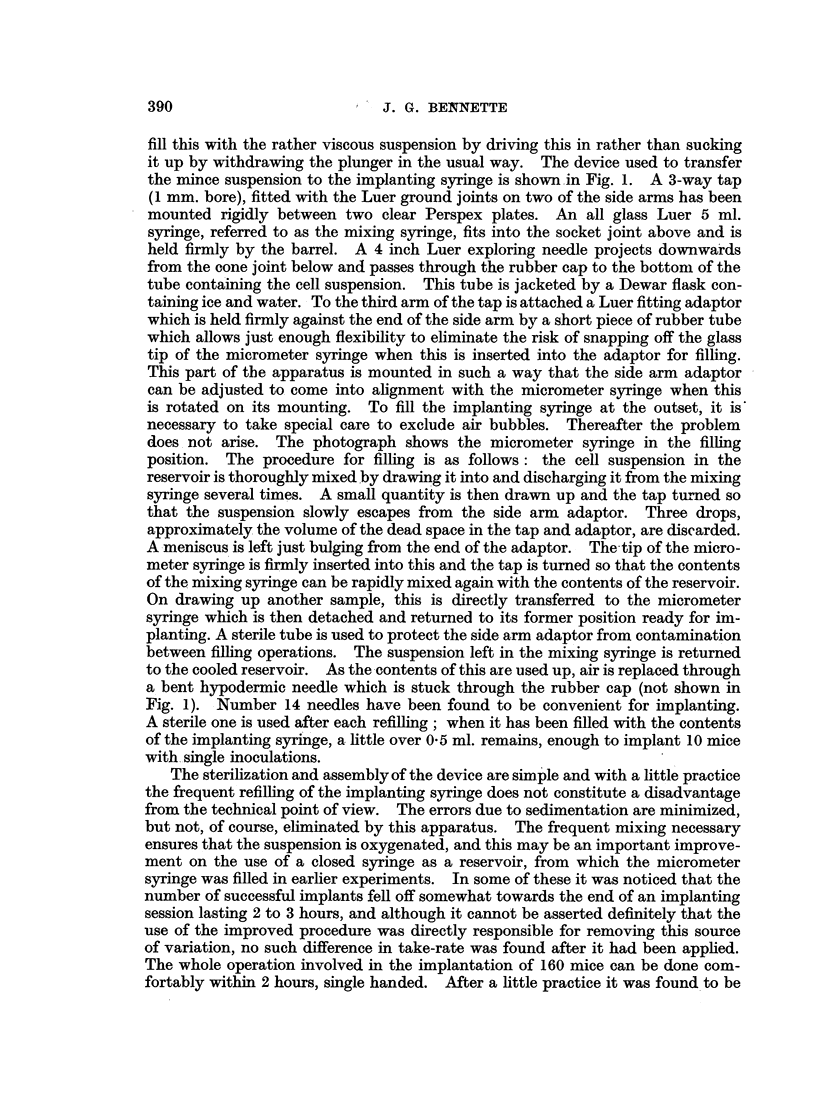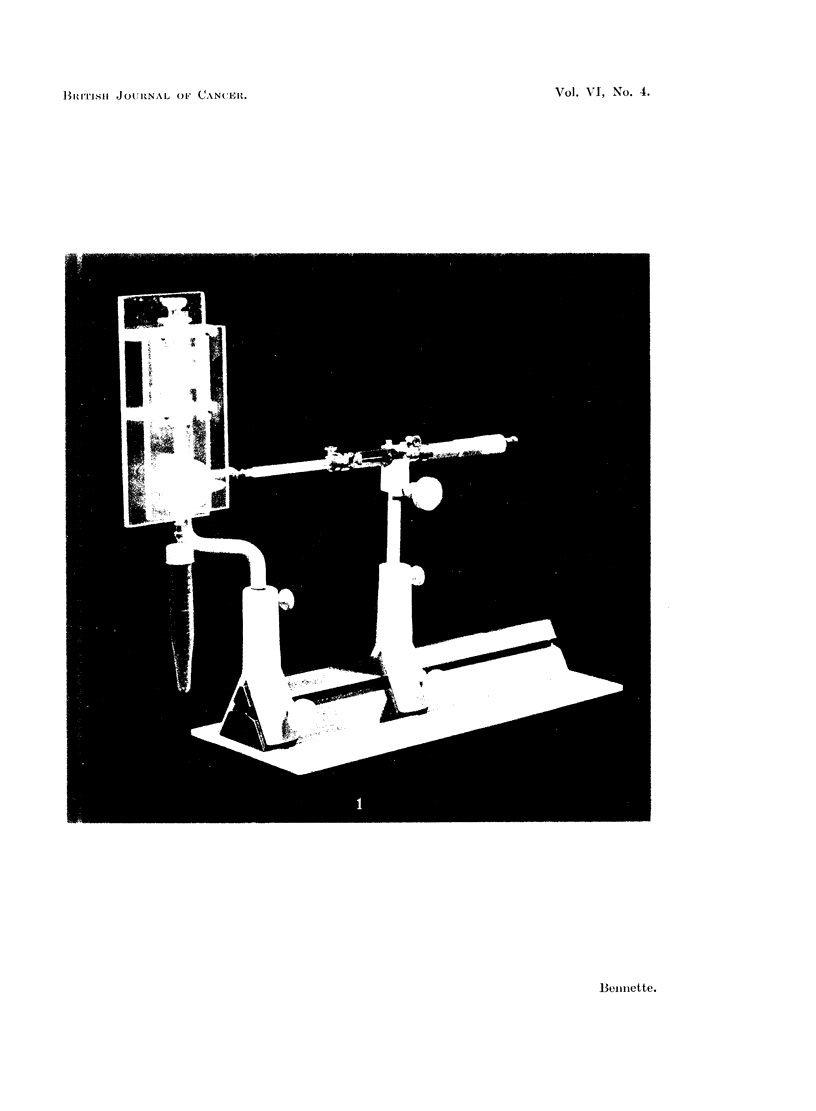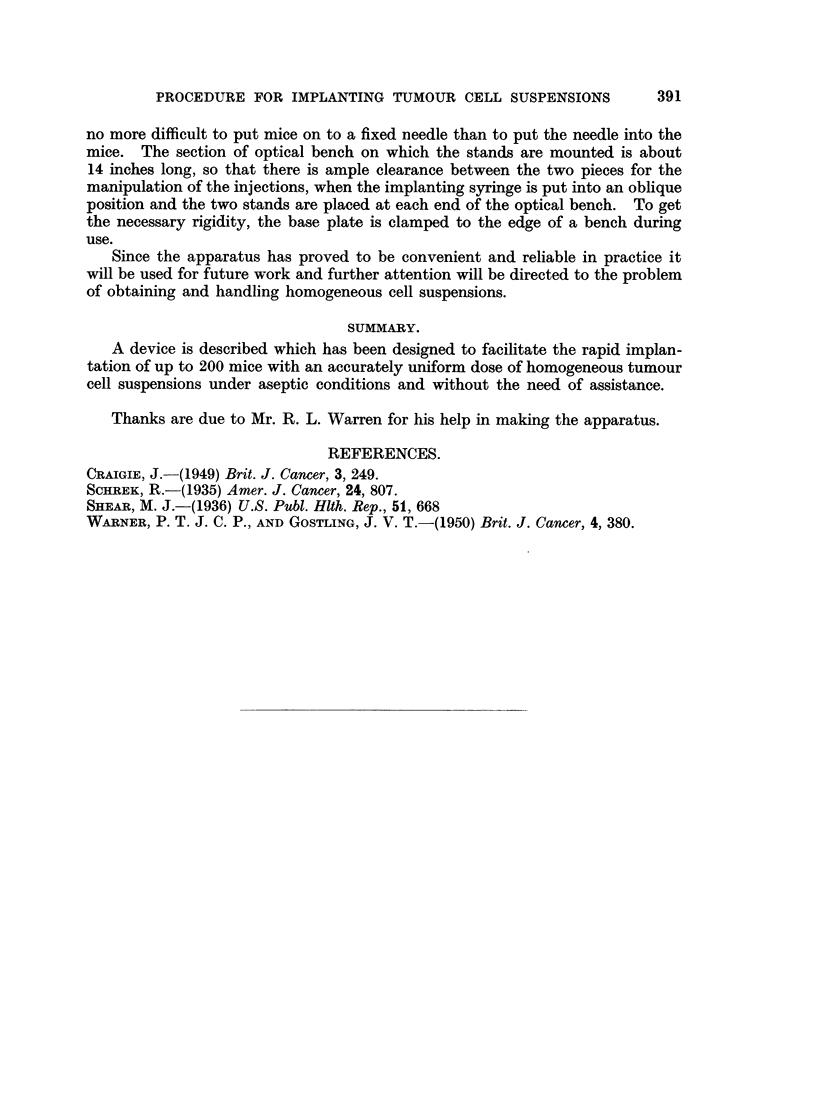# A Standard Procedure for Implanting Tumour Cell Suspensions

**DOI:** 10.1038/bjc.1952.43

**Published:** 1952-12

**Authors:** J. G. Bennette

## Abstract

**Images:**


### A STANDARD PROCEDURE FOR IMPLANTING TUMOUR CELL SUSPENSIONS.

J. G. BENNETTE.

*From the Courtauld Institute of Biochemistry, Middlesex Hospital, London, W.1.*

Received for publication October 14, 1952.

THE need to standardize the procedure for implanting tumours when quantitative work is being done is specially important in experiments involving the use of the titration method, by means of which the tumour-producing potency of a suspension of tumour cells is estimated (Schrek, 1935 ; Shear, 1936 ; Warner and Gostling, 1950). In work in which the growth rate of tumours is being studied it is also an advantage, at least theoretically, to base the information gained on the knowledge that the implantation was done by a uniform method. This is particularly so when tumours are implanted into pure strain animals in which their growth tends to be more uniform than in mixed strain animals.

To this end, use has been made of the Agla micrometer syringe (Burroughs Wellcome) which is designed to deliver accurately uniform volumes. With the help of a device to facilitate aseptic manipulation of the tumour cell suspensions, a technique has been developed which makes it possible to implant tumours rapidly and accurately without the need of assistance.

The procedure used for routine implanting at one dilution will be described. A mince is prepared using Craigie's pressure mincer (Craigie, 1949). There is some evidence (Epstein, M. A., personal communication) that the best results are obtained when two plungers of grooves of depths 0·5 and 0·125 mm. are used. Pieces of tumour for mincing are taken from the peripheral parts of large but not ulcerated tumours. The operation is done aseptically and samples of the mince are plated on to blood agar before and after the implantation. A volume of mince measured as accurately as possible from a syringe, is transferred to a sterile rubber capped graduated centrifuge tube and sterile 0·9 per cent (w/v) NaCl is added to make a 50 per cent (v/v) suspension, using the graduations of the tube. The suspension is then centrifuged at 1000 r.p.m. for 2 minutes to remove minute air bubbles which remain in the suspension even after the addition of the diluent.

The micrometer syringe is mounted horizontally and rigidly on a stand. The mounting can be swivelled through 90°, the movement being controlled by a stop and a tightening screw. The all glass syringe supplied with the instrument has not been used. It is rather fragile for use in this context and the nozzle has so fine a bore that blocking by fibrous material in the suspension proved to be troublesome. Furthermore, the high degree of accuracy of which it is capable seemed inappropriate for this kind of work. An ordinary good quality Luer tuberculin syringe has been calibrated and has been shown to be well suited for attachment to the micrometer fitting. It was found to be more satisfactory to fill this with the rather viscous suspension by driving this in rather than sucking it up by withdrawing the plunger in the usual way. The device used to transfer the mince suspension to the implanting syringe is shown in Fig. 1. A 3-way tap (1 mm. bore), fitted with the Luer ground joints on two of the side arms has been mounted rigidly between two clear Perspex plates. An all glass Luer 5 ml. syringe, referred to as the mixing syringe, fits into the socket joint above and is held firmly by the barrel. A 4 inch Luer exploring needle projects downwards from the cone joint below and passes through the rubber cap to the bottom of the tube containing the cell suspension. This tube is jacketed by a Dewar flask containing ice and water. To the third arm of the tap is attached a Luer fitting adaptor which is held firmly against the end of the side arm by a short piece of rubber tube which allows just enough flexibility to eliminate the risk of snapping off the glass tip of the micrometer syringe when this is inserted into the adaptor for filling. This part of the apparatus is mounted in such a way that the side arm adaptor can be adjusted to come into alignment with the micrometer syringe when this is rotated on its mounting. To fill the implanting syringe at the outset, it is necessary to take special care to exclude air bubbles. Thereafter the problem does not arise. The photograph shows the micrometer syringe in the filling position. The procedure for filling is as follows: the cell suspension in the reservoir is thoroughly mixed by drawing it into and discharging it from the mixing syringe several times. A small quantity is then drawn up and the tap turned so that the suspension slowly escapes from the side arm adaptor. Three drops, approximately the volume of the dead space in the tap and adaptor, are discarded. A meniscus is left just bulging from the end of the adaptor. The tip of the micrometer syringe is firmly inserted into this and the tap is turned so that the contents of the mixing syringe can be rapidly mixed again with the contents of the reservoir. On drawing up another sample, this is directly transferred to the micrometer syringe which is then detached and returned to its former position ready for implanting. A sterile tube is used to protect the side arm adaptor from contamination between filling operations. The suspension left in the mixing syringe is returned to the cooled reservoir. As the contents of this are used up, air is replaced through a bent hypodermic needle which is stuck through the rubber cap (not shown in Fig. 1). Number 14 needles have been found to be convenient for implanting. A sterile one is used after each refilling ; when it has been filled with the contents of the implanting syringe, a little over 0·5 ml. remains, enough to implant 10 mice with single inoculations.

The sterilization and assembly of the device are simple and with a little practice the frequent refilling of the implanting syringe does not constitute a disadvantage from the technical point of view. The errors due to sedimentation are minimized, but not, of course, eliminated by this apparatus. The frequent mixing necessary ensures that the suspension is oxygenated, and this may be an important improvement on the use of a closed syringe as a reservoir, from which the micrometer syringe was filled in earlier experiments. In some of these it was noticed that the number of successful implants fell off somewhat towards the end of an implanting session lasting 2 to 3 hours, and although it cannot be asserted definitely that the use of the improved procedure was directly responsible for removing this source of variation, no such difference in take-rate was found after it had been applied. The whole operation involved in the implantation of 160 mice can be done comfortably within 2 hours, single handed. After a little practice it was found to be

1

Bennette.

no more difficult to put mice on to a fixed needle than to put the needle into the mice. The section of optical bench on which the stands are mounted is about 14 inches long, so that there is ample clearance between the two pieces for the manipulation of the injections, when the implanting syringe is put into an oblique position and the two stands are placed at each end of the optical bench. To get the necessary rigidity, the base plate is clamped to the edge of a bench during use.

Since the apparatus has proved to be convenient and reliable in practice it will be used for future work and further attention will be directed to the problem of obtaining and handling homogeneous cell suspensions.

### SUMMARY.

A device is described which has been designed to facilitate the rapid implantation of up to 200 mice with an accurately uniform dose of homogeneous tumour cell suspensions under aseptic conditions and without the need of assistance.

Thanks are due to Mr. R. L. Warren for his help in making the apparatus.

### REFERENCES.

Craigie, J.—(1949) *Brit. J. Cancer*, **3**, 249.
Schrek, R.—(1935) *Amer. J. Cancer*, **24**, 807.
Shear, M. J.—(1936) *U.S. Publ. Hlth. Rep.*, **51**, 668
Warner, P. T. J. C. P., and Gostling, J. V. T.—(1950) *Brit. J. Cancer*, **4**, 380.